# Supplementary material for: Identification of large offspring syndrome during pregnancy through ultrasonography and maternal blood transcriptome analyses
Source: Sci Rep. 2022 Jun 22;12:10540. doi: 10.1038/s41598-022-14597-w (PMC9217928; doi:10.1038/s41598-022-14597-w)
Supplement: Supplementary file 1 — Supplementary Figures. [file 41598_2022_14597_MOESM1_ESM.pdf]

Identification of large offspring syndrome during pregnancy through ultrasonography and maternal blood transcriptome analyses

Rocío Melissa Rivera et al., 2022. Scientific Reports

Supplemental Figures

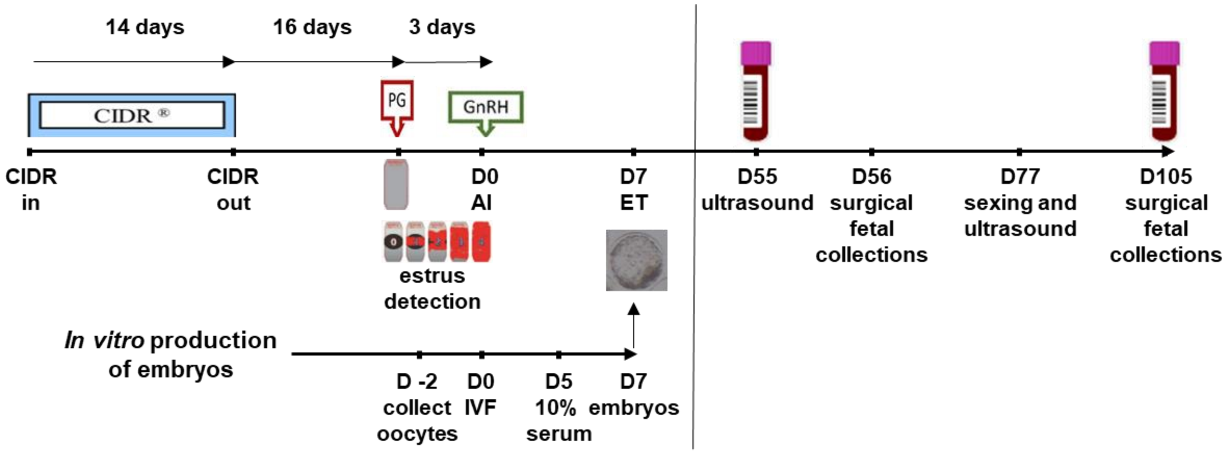

Supplemental Figure 1. Experimental design. Production of day 56 and day 105 fetuses.

For estrus synchronization, the 14-day CIDR®- PG & TAI protocol was followed. Briefly, CIDRs were placed in the heifers and removed 14 days later. Sixteen days after CIDR removal, 25 milligram of prostaglandin F2 alpha (Lutalyze); Zoetis, NJ) was administered intramuscularly. Concurrent with the administration of prostaglandin F2 alpha, a breeding indicator patch (Estrotect, Genex, Shawano, WI) was applied to each animal across the backbone as per the manufacturer's instructions. Estrus was checked three times per day (7:00 h, 12:00 h and 16:00 h/17:00 h) for three consecutive days and only animals with a heat score two and above at the time of artificial insemination (AI; 13:00 h) were selected for AI or embryo transfer (ET). The breeding indicators were scored 0-4, with a score of 0 indicating no patch activation; a score of 1 signifying <25% patch activation; a score of 2 signifying >25 to <50% patch activation; a score of 3 signifying >50 to <75% patch activation; and a score of 4 signifying >75% patch activation. Heifers were randomly assigned to the AI or ET group. Regardless of experimental group, all animals were injected with 100 microgram of gonadotropin releasing hormone (GnRH) intramuscularly (Factrel, Zoetis) at the time of corresponding to the insemination in the AI group. In order to collect the number of fetuses required for the experiment, two sets of estrus synchronizations were performed (in November of 2018 and in February of 2019).

In vitro production (IVP) of bovine embryos was done simultaneously with estrus synchronization to ensure AI (control) and IVP embryos were of the same chronological age on day 7 after estrus. Media and procedures were as previously described by us. Briefly, Bos *taurus taurus* (*B. t. taurus*; Angus/Angus-Crossbred) ovaries were obtained at an abattoir and oocytes collected at Oklahoma State University (OSU) in Stillwater Oklahoma. Oocytes were placed in CO_2 equilibrated maturation medium and shipped overnight at 38.5°C to the

University of Missouri (MU) or the University of Florida (UF). In addition, *B. t. taurus* oocytes were also purchased from DeSoto Biosciences (Seymour, TN, USA) and processed at MU. All media for embryo production were prepared at MU by a single technician and shipped overnight to the pertinent location prior to each procedure. Two sources of oocytes and IVP locations were used to ensure sufficient embryos were available for embryo transfer in case of technical or weather-related difficulties. Oocytes were removed from maturation medium after ~21 h of culture and inseminated with semen from one *B. t. indicus* male (Brahman breed [JDH MR MANSO 7 960958 154BR599 11200 EBS/INC CSS 2]). Putative zygotes were stripped of cumulus cells by five minutes vigorous vortexing at approximately 18 h after insemination and cultured in KSOM supplemented with amino acids in a humidified atmosphere containing 5% O2, 5% CO2, and 90% N2. On day five after insemination, the culture medium was supplemented with 10% (v/v) estrus cow serum (collected and prepared in house and previously used in 2) and embryos returned to the incubator. Day 6 embryos produced at UF were shipped overnight at 38.5°C in serum supplemented culture medium to MU. On day seven, blastocyst-stage IVP embryos were selected, washed in BioLife Holding & Transfer Medium (AgTech; Manhattan, KS), and loaded in groups of two into 0.25 cc yellow, direct transfer and irradiated straws (AgTech) and kept in a Styrofoam box until ET. Blastocysts were transferred to synchronized recipient females on day seven after GnRH injection

Maternal blood was collected on D55 and D105 of gestation.

CIDR: controlled internal drug release, an intravaginal progesterone releasing device. PG: prostaglandin. GnRH: gonadotropin releasing hormone. AI: artificial insemination. IVF: *in vitro* fertilization. D0: day of AI or IVF.

Selection of dams for transcriptome analysis

AI

Fetal Sex	F	F	F	M	M	F	M	M
Number of fetuses	1	1	1	1	1	1	1	1
Weight of fetus/es	396	388	414	442	466	468	544	550

IVP N

Fetal Sex	F	F	M	M	F, M	M, M
Number of fetuses	1	1	1	1	2	2
Weight of fetus/es	408	442	480	538	444 & 448 (F, M)	434 & 532 (M, M)

IVP LOS

Fetal Sex	M	F	F	M	F	M	F, M	M	M, F
Number of fetuses	1	1	1	1	1	1	2	1	2
Weight of fetus/es	586	638	578	648	704	752	506 & 556 (F,M)	1080	584 & 986 (M & F)

Supplemental Figure 2. Information on the dams used for WBC transcriptome analyses. AI = artificial insemination (i.e. control). IVP-N = embryos were produced by *in vitro* procedures and were <97% of the control's weight at D105. IVP-LOS embryos were produced by in vitro procedures and were ≥97% of the control's weight at D105. F = female. M = male.

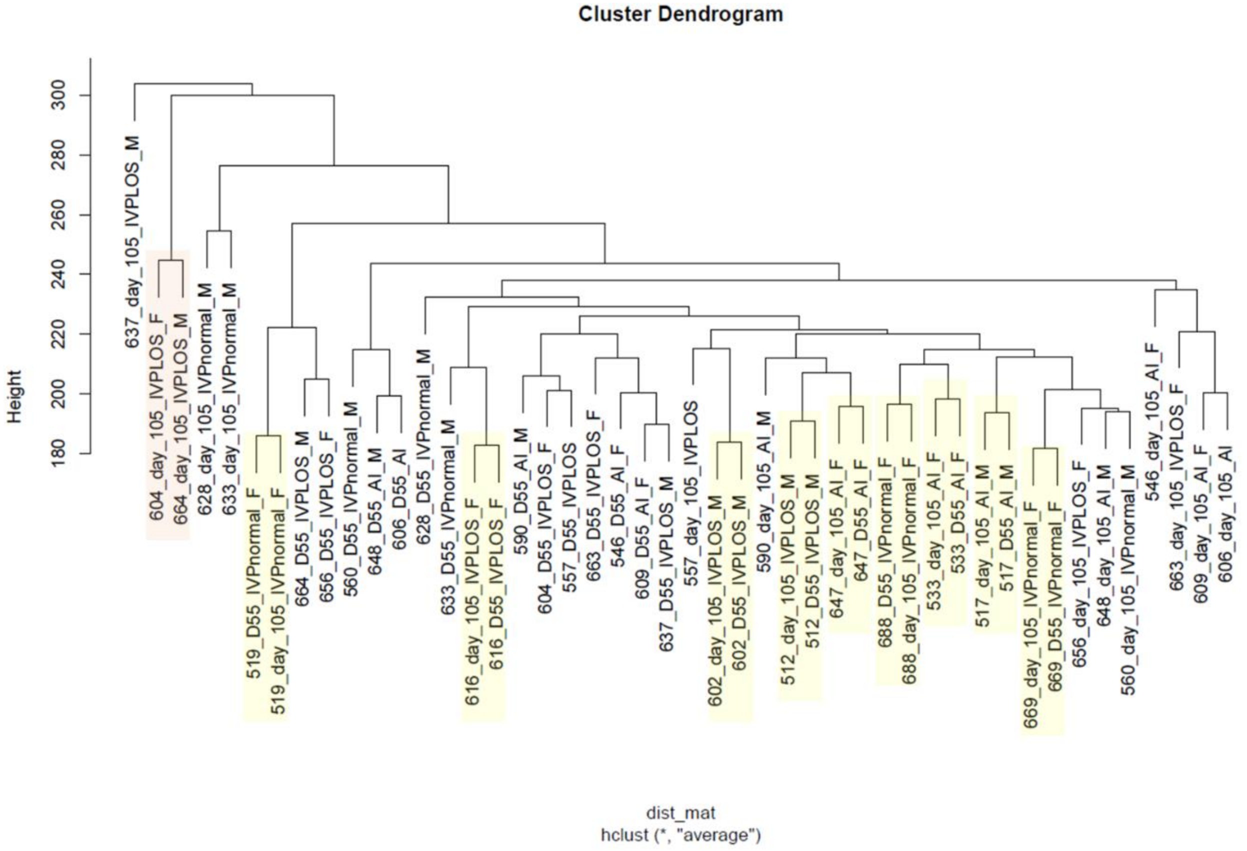

Supplemental Figure 3. Unsupervised Hierarchical Clustering. Samples highlighted in orange are the WBC transcriptomes of the females carrying the two largest LOS (604 and 664). Samples highlighted in yellow clustered by female irrespective of the blood having been collected on D55 and D105 of pregnancy and during winter and summer respectively. AI = artificial insemination (i.e. control). IVPnormal = embryos were produced by *in vitro* procedures and were <97% of the control's weight at D105. IVP-LOS embryos were produced by in vitro procedures and were ≥97% of the control's weight at D105. F = female. M = male.

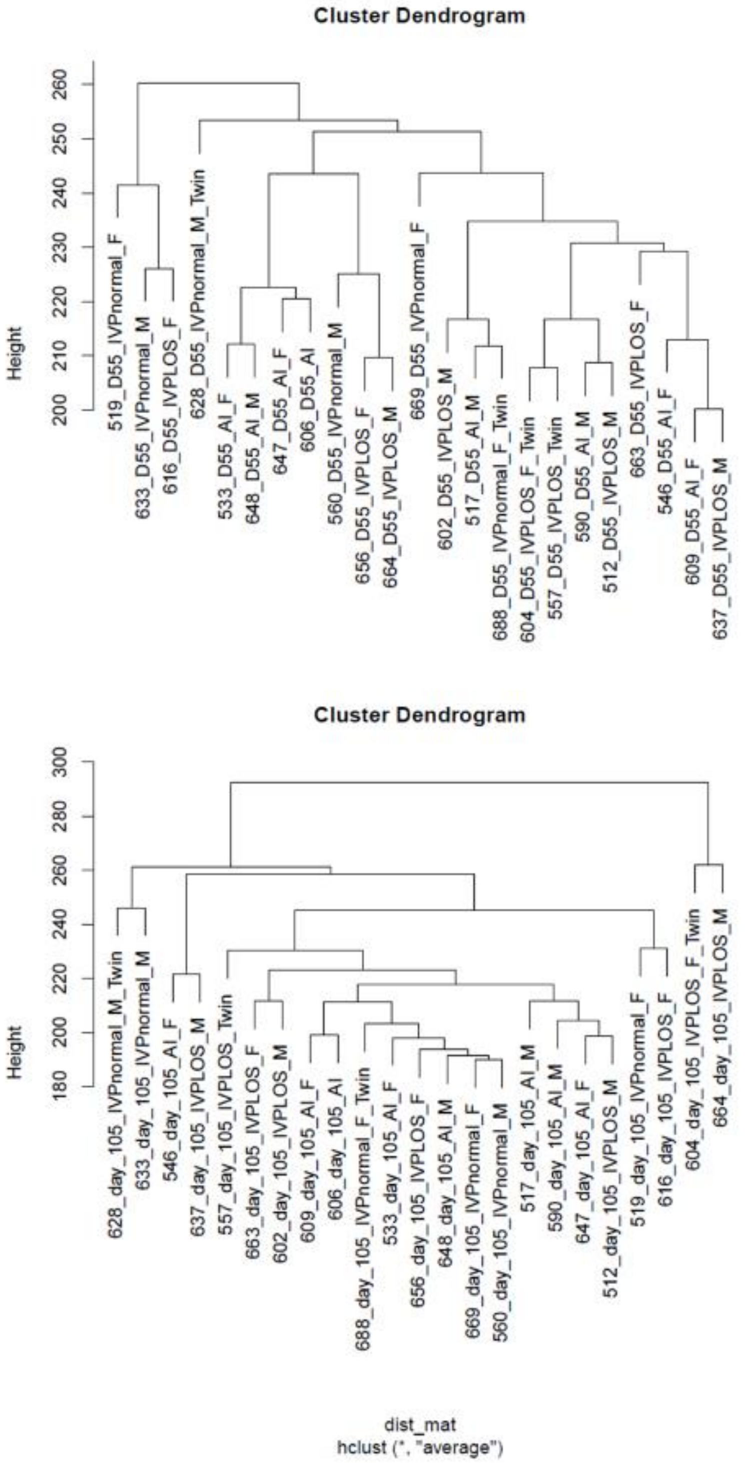

Supplemental Figure 4. Unsupervised Hierarchical Clustering for D55 and D105 samples.

Labels as above.
